# Characterization of Schistosome Tegumental Alkaline Phosphatase (SmAP)

**DOI:** 10.1371/journal.pntd.0001011

**Published:** 2011-04-05

**Authors:** Rita Bhardwaj, Patrick J. Skelly

**Affiliations:** Molecular Helminthology Laboratory, Division of Infectious Diseases, Department of Biomedical Sciences, Cummings School of Veterinary Medicine, Tufts University, Grafton, Massachusetts, United States of America; George Washington University Medical Center, United States of America

## Abstract

Schistosomes are parasitic platyhelminths that currently infect over 200 million people globally. The parasites can live for years in a putatively hostile environment - the blood of vertebrates. We have hypothesized that the unusual schistosome tegument (outer-covering) plays a role in protecting parasites in the blood; by impeding host immunological signaling pathways we suggest that tegumental molecules help create an immunologically privileged environment for schistosomes. In this work, we clone and characterize a schistosome alkaline phosphatase (SmAP), a predicted ∼60 kDa glycoprotein that has high sequence conservation with members of the alkaline phosphatase protein family. The SmAP gene is most highly expressed in intravascular parasite life stages. Using immunofluorescence and immuno-electron microscopy, we confirm that SmAP is expressed at the host/parasite interface and in internal tissues. The ability of living parasites to cleave exogenous adenosine monophosphate (AMP) and generate adenosine is very largely abolished when SmAP gene expression is suppressed following RNAi treatment targeting the gene. These results lend support to the hypothesis that schistosome surface enzymes such as SmAP could dampen host immune responses against the parasites by generating immunosuppressants such as adenosine to promote their survival. This notion does not rule out other potential functions for the adenosine generated e.g. in parasite nutrition.

## Author Summary

Schistosomes are parasitic worms that live for many years in the blood stream of ∼200 million people globally. How they manage to avoid getting killed by host immune mechanisms is a puzzle. We hypothesize that molecules in their skin (tegument), e.g. the enzyme alkaline phosphatase (SmAP), help them in this regard. In this work, we characterize SmAP. It is predicted to be a ∼60 kDa glycoprotein that is most highly expressed in those parasite life stages that live inside a mammalian host. We confirm that SmAP is expressed at the host/parasite interface as well as in internal tissues. To monitor the function of the enzyme, we have suppressed the expression of the SmAP gene using RNA interference. These parasites largely loose their ability to cleave the added substrate AMP (adenosine monophosphate) to generate adenosine. Since adenosine is very efficient at dampening host immune mechanisms, our work lends support to the idea that one function for tegumental SmAP is to generate the immunosuppressant adenosine so that the worms can remain unperturbed by host immune effectors. Our results do not preclude other functions for the generated adenosine. For example it could also be taken up as a nutrient by the parasites.

## Introduction

Schistosomes are parasitic platyhelminths that constitute an important public health problem globally. Infection can lead to a chronic, often debilitating disease that afflicts more than 200 million people in over 70 countries worldwide [1], [2]. Mortality is estimated at over a quarter of a million deaths annually in sub-Saharan Africa alone, with many millions more experiencing chronic morbidity [1], [2]. Schistosome infection is characterized by the presence of relatively large adult worms (blood flukes) within the vasculature of their hosts. Schistosomes in the bloodstream are surrounded by all the components of immunity, yet remain able to survive, sometimes for decades. This means that they must possess evasion strategies that can overcome anti-worm immune mechanisms. While parasite eggs laid by female worms are vigorously targeted by the host immune response, there is no overt cellular inflammation detected around the adult worms themselves in the vasculature [3]. This is the case despite the fact that the adult parasites are relatively big and possess a large interface with the host. One major interface between the schistosome and its external environment is the tegument. This is a unique, syncytial structure that is bounded externally by a dual lipid bilayer [4], [5], [6], [7]. This double-bilayered (or heptalaminate) membrane is unique to blood-dwelling trematodes such as schistosomes and is not found in trematode parasites occupying other habitats [8]. We are interested in understanding how this structure contributes to schistosome success. It is clear that the tegument surface contains molecules that perform vital functions such as nutrient uptake [9], [10]. Less clear is how, or if, the structure contributes to the ability of the worms to avoid immune elimination. In this report we focus on the schistosome surface alkaline phosphatase (SmAP) a protein which we hypothesize plays a role in immune evasion by generating the potent immunosuppressant adenosine. Since adenosine is also an important nutrient, SmAP may also play a role in parasite feeding.

## Methods

### Ethics Statement

Infection of mice with schistosome parasites was carried out following review and approval by the Institutional Animal Care and Use Committee of Tufts University. The Tufts animal management program is accredited by the American Association for the Accreditation of Laboratory Animal Care, meets the National Institutes of Health standards as set forth in the “Guide for the Care and Use of Laboratory Animals” (National Academy Press, Washington DC, 1996), and accepts as mandatory the PHS “Policy on Humane Care and Use of Laboratory Animals by Awardee Institutions” and NIH “Principals for the Utilization and Care of Laboratory Animals Used in Testing, Research and Training”.

### Parasites

*Biomphalaria glabrata* snails infected with the Puerto Rican strain of *S. mansoni* were obtained from Dr. Fred Lewis (Biomedical Research Institute, Rockville, MD). Schistosomula were prepared from cercariae released from infected snails and were cultured in Basch medium (lacking red blood cells (rbcs)) at 37°C, in an atmosphere of 5% CO_2_ as described [11]. Adult worms were recovered by vascular perfusion from Balb/c mice that were infected with 125 cercariae, 7 weeks previously. Adult worms were maintained in Basch medium (lacking rbcs). Parasite eggs were isolated from infected mouse liver tissue, miracidia were recovered, and sporocysts were prepared and cultured for 24 h, as described previously [12].

### Cloning SmAP

An expressed sequence tag (EST) encoding a fragment of a putative *S. mansoni* alkaline phosphatase (EST269800) was identified in the adult female cDNA library of Phil LoVerde/Joe Merrick (GenBank accession number AI975206). Using this information, we designed two specific SmAP oligonucleotides for use with the Smart RACE cDNA amplification kit (Clontech, CA) to amplify the entire coding region. The oligonucleotides were synthesized based on sequence of the predicted 3′untranslated region of the EST. The 3′-most specific oligonucleotide (AP-3: 5′-GTAATATTTATTAGGTTAGATTAG-3′) was used to synthesize cDNA. The second oligonucleotide (AP-4: 5′-CATGTTAATCATGTAAGTATATTT-3′) was used, together with the Smart RACE kit anchor primer and the cDNA, in a semi-nested PCR following the manufacturer's instructions, in order to amplify the complete SmAP coding DNA presented here. The ∼1.5 kb PCR product was excised from an agarose gel using a GeneClean kit (Bio101, CA) and sequenced.

### Anti-SmAP antibody production

**Figure 1:**
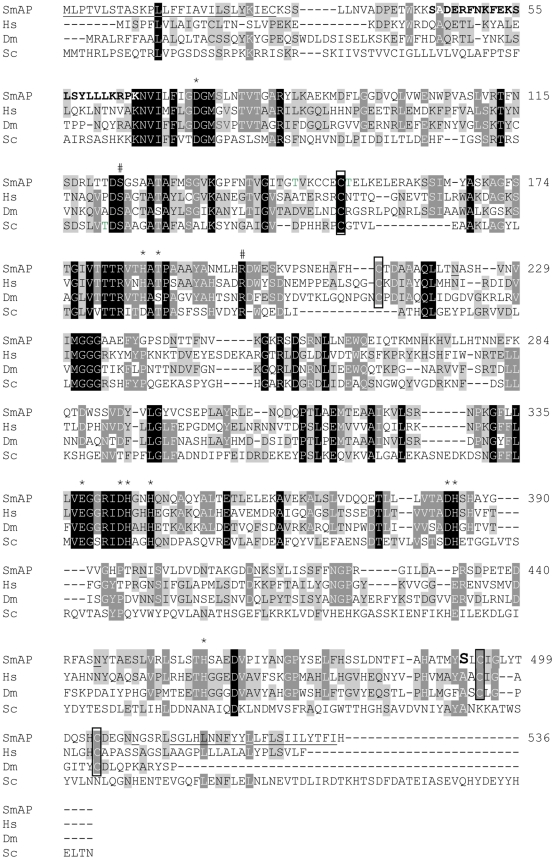
Alignment of SmAP predicted amino acid sequence with homologs. Designations and GenBank accession numbers of these proteins are: SmAP, *Schistosoma mansoni* (EU040139); Hs, *Homo sapiens* (NP000469); Dm, *Drosophila melanogaster* (NP649315) and Sc, *Saccharomyces cerevisiae* (AAA34871). Identical residues are indicated by shading. Proposed active site residues are indicated by # and residues reported to be important in metal binding are indicated by *. A potential signal peptide sequence at the N-terminal end and a single predicted transmembrane domain at the C-terminus are both underlined. A potential site for GPI-linked modification is S^492^, indicated in bold. Conserved cysteine residues are boxed. The peptide sequence from residue 44 to 65, used to generate anti-SmAP antibodies, is indicated in bold. Underlined asparagine (N) residues indicate several potential N-linked glycosylation sites.

A peptide comprising SmAP amino acid residues 44–65 (NH_2_-SADERFNKFEKSLSYLLLKRPK-COOH) was synthesized by Genemed Synthesis, Inc., TX. This sequence is indicated in bold script in Fig. 1. A cysteine residue was added at the amino-terminus to facilitate conjugation of the peptide to bovine serum albumin (BSA). Approximately 500 µg of the peptide-BSA conjugate in Freund's Complete Adjuvant was used to immunize a New Zealand white rabbit subcutaneously. The rabbit was boosted with 100 µg peptide alone in Incomplete Freund's Adjuvant 20, 40 and 60 days later. Ten days following this, serum was recovered and anti-SmAP antibodies were affinity purified using a peptide-ovalbumin conjugate and dialysed against phosphate buffered saline (PBS), as previously described [13].

### SmAP gene expression analysis

The levels of expression of the SmAP gene in different life cycle stages was measured by quantitative real time PCR (qRT-PCR) using the housekeeping gene triose phosphate isomerase as the endogenous control [14]. In unpublished work we have identified a greater uniformity in expression of the triose phosphate isomerase gene (TPI) between different schistosome life stages and we use this as the endogenous control in the developmental expression work presented here. The procedure, involving total RNA extraction and qRT-PCR, has been described [14]. Briefly, RNA was first extracted using the Trizol method (Invitrogen, CA) and residual DNA was removed by DNase digestion using a TurboDNA-free kit (Applied Biosystems, TX). cDNA was synthesized using 1 µg RNA, an oligo (dT)_20_ primer and Superscript III RT (Invitrogen, CA).

Quantitative real time PCR was performed using custom TaqMan Assays using primer sets and reporter probes labeled with 6-carboxyfluorescein (FAM), obtained from Applied Biosystems, CA. To detect SmAP expression the following primers and probe were used: SmAP1Taq-siteF, 5′-GCCATCCGACAAGGAATATAAGTGT-3′; SmAP1Taq-siteR, 5′-GGTCCATTGAAAAAGGAGGATATGAGA-3′and probe, SmAP1Taq-siteM2, 5′-FAM-ATCTCCTTTTGCAGTATTATC-3′. Each real-time TaqMan PCR was performed using cDNA equivalent to 10 ng total parasite RNA according to the manufacturer's universal conditions PCR protocol, in a final volume of 25 µl. All samples were run in triplicate and underwent 40 amplification cycles on a 7500 ABI PRISM Sequence Detection System Instrument. For relative quantification, the ΔΔCt method was employed [15]. Data shown are representative of at least 3 replicate experiments.

### Membrane preparation, gel electrophoresis and western blotting analysis

Membrane extracts from different parasite life stages were prepared [16] and aliquots from each were resolved by 4–15% gradient SDS-PAGE. One gel was stained with Coomassie Blue and protein from a duplicate gel was blotted to PVDF membrane, as previously outlined [13]. Blots were probed with purified anti-SmAP antibodies at 1∶300 dilution and bound antibody was detected using a horseradish peroxidase-labeled anti-rabbit IgG (1∶5000, GE Healthcare, NJ) and the TMB Membrane Peroxidase system from Kirkegaard and Perry Laboratories Inc, MD, following the manufacturer's instructions. Blot images were captured using a Kodak Image Station 2000RT.

In one experiment, the reducing agent dithiothreitol (DTT) was omitted from the gel electrophoresis sample buffer prior to protein resolution by SDS-PAGE. One lane from such a gel was incubated overnight in alkaline phosphatase substrate (1 mg/ml 5-Bromo-4-Chloro-3-Indolyl-Phosphate (BCIP) in 50 mM Tris HCl, pH 7.6) to visualize protein bands displaying enzymatic activity[17]. A second lane was blotted to PVDF membrane and processed for western blotting analysis, as outlined in the preceding paragraph.

### Immunolocalization

Adult worm sections 7 µm thick were obtained using a cryostat and fixed in cold acetone. Immunofluorescent detection of SmAP was carried out using affinity-purified, rabbit anti-SmAP antiserum diluted 1∶20 and Alexa fluor 488-conjugated goat anti-rabbit IgG (Invitrogen, CA), essentially as described earlier [18].

### Immunogold Labeling and Electron Microscopy

Freshly perfused adult parasites were fixed overnight with 2% glutaraldehyde in 0.1 M cacodylate buffer at 4°C. The samples were then dehydrated in a graded series of ethanol, then infiltrated and embedded in L.R. white acrylic resin. Ultramicrotomy was performed using a Leica Ultracut R ultramicrotome and the sections collected on gold grids. Grids were immunolabeled in a two step method according to the following procedure; the grids were conditioned in PBS for 5 min×3 at room temperature (RT), followed by the blocking of non-specific labeling for 30 min at RT using 5% non-fat dry milk in PBS. After rinsing, the grids were exposed to primary antibody diluted 1∶30 for 1 hour at RT, followed by washing in PBS and then incubated with secondary antibody diluted 1∶30 (10 nm gold-labeled goat anti-rabbit IgG (H&L, GE Healthcare, NJ)) for 1 hour at RT, and finally rinsed thoroughly in water. The grids were exposed to osmium vapor and/or lightly stained with lead citrate to improve contrast and were examined and photographed using a Philips CM 10 electron microscope at 80 KV.

### Treatment of parasites with siRNAs

Adult worms were treated either with a synthetic siRNA targeting SmAP and designated SmAPsiRNA 1 (5′-AAGAAATCAGCAGATGAGAGATTTAAT-3′) or with control siRNAs. Control siRNAs were of two forms, the first targeted an unrelated schistosome gene (encoding a phosphodiesterase, GenBank accession number EU769293; Sm control; 5′-TTGATGGATTTCGTTATGATTACTTTG-3′) and the second targeted no sequence in the schistosome genome. The sequence of this “irrelevant control” is 5′-CTTCCTCTCTTTCTCTCCCTTGTGA-3′. Delivery of siRNAs to the parasites was performed by electroporation as described previously, using 10 µg of each siRNA [14], [19]. Gene suppression was assessed post-treatment by comparing mRNA and protein levels in target versus control groups.

To assess the level of suppression post-siRNA treatment, RNA and protein were isolated from worm lysates (PARIS kit, Applied Biosystems, CA). To monitor SmAP levels at the RNA level, the same qRT-PCR protocol described above was employed but here using the alpha tubulin gene as the endogenous control for each sample, as previously [15]. For graphical representation, the ΔΔCt values were normalized to controls and expressed as percent difference [15]. Results were graphed as gene expression level relative to the group treated with control irrelevant siRNA. To monitor SmAP levels at the protein level, western blotting analysis was carried out essentially as described previously [20]. To monitor protein loading per lane, a duplicate gel was stained with Coomassie Brilliant Blue.

### Alkaline phosphatase activity of live adult worms

Eight days after RNAi treatment, the alkaline phosphatase activity of live adult worms was assessed by culturing 6 SmAP-suppressed and control parasites in triethanolamine buffer, pH 8.9, containing chromogenic substrate p-nitrophenyl phosphate (p-NPP, Sigma-Aldrich, MO) at 37°C in a final volume of 1 ml. After 30 min incubation, 400 µl of culture supernatant was recovered and read at OD 405 nm. Buffer containing substrate alone served as a blank.

### AMP degradation and adenosine generation by live adult worms

Eight days after RNAi treatment, 12 adult male SmAP-suppressed and control parasites were incubated at 37°C in RPMI medium lacking fetal calf serum and containing 500 µM AMP (Sigma-Aldrich, MO). Samples (70 µl) of culture media were withdrawn at selected time points and the levels of AMP and adenosine they contained was measured using high performance liquid chromatography (HPLC). HPLC assays was performed by loading samples onto a strong anion-exchange partisphere SAX column (Whatman/GE Healthcare, NJ) with a linear gradient from 7 mM KH_2_PO_4_, pH 3.8 to 0.5 M KH_2_PO_4_, pH 4.5 for 60 min and a flow rate of 0.5 ml/min [21]. For standardization, purified AMP and adenosine (Sigma-Aldrich, MO) in RPMI were first applied to the column and their characteristic elution profiles determined.

### Statistical analysis

The student's *t*-test was used to compare the means between a target group and a control group and *p* values less than 0.05 were considered significant.

## Results

### Sequence analysis

The cloned SmAP DNA potentially encodes a 536 amino acid protein of 59,375 Da and a pI of 5.92. The GenBank accession number for SmAP is EU040139. An alignment of this protein with other members of the alkaline phosphatase family using CLUSTALW is shown in Fig. 1 and several conserved motifs are highlighted. The designations of the compared proteins are as follows: Hs, *Homo sapiens*, tissue non-specific alkaline phosphatase; Dm, *Drosophila melanogaster*; Sc, the yeast *Saccharomyces cerevisiae*. SmAP exhibits a higher percent sequence identity with other animal alkaline phosphatases (33–34% versus human and *D. melanogaster* homologs) compared to the yeast enzyme (22%). In SmAP, active site residues S^123^ and R^198^ are highly conserved (indicated by #, Fig. 1) as are residues reported to be important in metal binding (D^73^, H^185^, T^187^, E^338^, D^343^, H^344^, H^347^, D^384^, H^385^, H^459^, indicated by *). The 28 amino acid, N-terminal, sequence (^1^MLPTVLSTASKPLLFFIAVILSLYKIEC^28^) is a potential signal peptide (using SignalP 3.0, http://www.cbs.dtu.dk/services/SignalP-3.0/), and this sequence is underlined. The protein has a single predicted transmembrane domain at the carboxyl terminus, ^514^SGLHLNNFYYLLFLSIILYTFI^535^, underlined in Fig. 1. The best potential site for GPI-linked modification is S^492^ (bold, Fig. 1), as determined at http://mendel.imp.ac.at/gpi/gpi_server. Conserved cysteine residues are boxed. The peptide ^44^SADERFNKFEKSLSYLLLKRPK^65^, used to generate anti-SmAP antibodies is in bold. Underlined asparagine (N) residues indicate several potential N-linked glycosylation sites.

### Developmental expression

**Figure 2:**
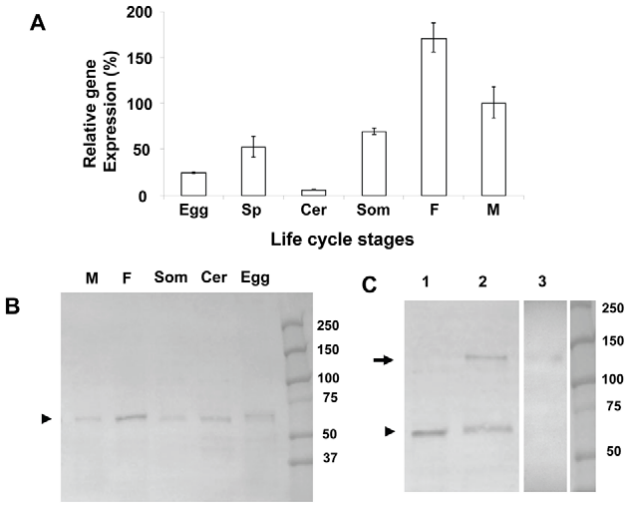
SmAP detection and expression. A. Expression of the SmAP gene in different schistosome developmental stages. Egg, sporocyst (Sp), cercaria (Cer), 24 h-cultured schistosomulum (som), adult female (F) and adult male (M), B. SmAP protein expression in membrane preparations from different schistosome developmental stages. Adult male (M), adult female (F), 24 h-cultured schistosomulum (Som), cercaria (Cer) and egg. The arrowhead indicates the position of the SmAP protein. Molecular mass markers are depicted in the right lane and sizes are indicated in kDa. C. Adult male and female worm extract was resolved in the presence (lane 1) or absence (lanes 2 and 3) of reducing agent by SDS-PAGE. Lanes 1 and 2 represent western blots of the extract probed with anti-SmAP antibodies. The arrowhead indicates the position of the SmAP monomeric protein (at ∼60 kDa) while the arrow indicates the proposed homodimeric form (running at ∼120 kDa). Lane 3 represents one tract of the gel after incubation with the alkaline phosphatase substrate BCIP. The arrow indicates a band of activity at ∼120 kDa.

The developmental expression of SmAP was examined in several life stages by qRT-PCR and results are shown in Fig. 2A. Gene expression is clearly detected in all life stages examined and relative expression is highest in the intra-mammalian life stages (schistosomula and adults).

In schistosome protein extracts, anti-SmAP antibodies detect a prominent band (arrowhead, Fig. 2B), running at the predicted size of SmAP (∼60 kDa) by western analysis in all life stages examined, namely: eggs, cercariae, schistosomula and adult male and female worms, when extracts are resolved in the presence of reducing agent. The adult membrane preparation was also resolved either in the presence (Fig. 2C, lane 1) or absence (Fig. 2C, lane 2) of the reducing agent DTT. In the absence of this reagent, an additional ∼120 kDa species is detected in the extract which may represent the SmAP homodimer (arrow, Fig. 2C, lane 2). This dimeric form is predicted to be the functional enzyme. Indeed, in gel slices incubated with alkaline phosphatase substrate (BCIP), reaction product is detected at the same position (∼120 kDa, arrow, Fig. 2C, lane 3).

### Localization of SmAP in adult tissues

**Figure 3:**
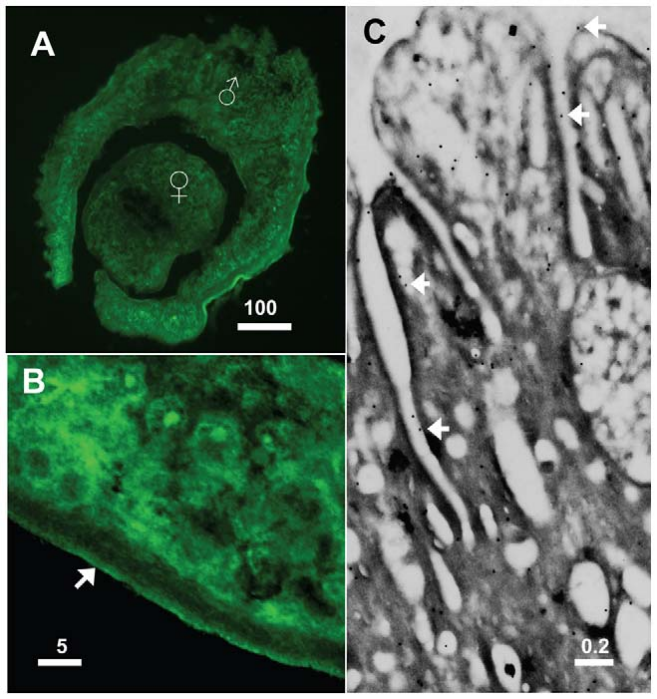
Immunolocalization of SmAP in adult parasites. A. Cross section through a male/female couple showing widespread immunofluorescent staining with anti-SmAP antibody. B. Higher magnification image of the peripheral tissue of an adult male. The arrow indicates the outer tegument. C. Electron micrograph of the adult tegument showing immunogold labeling of SmAP. Arrows indicate gold particles at the host-parasite interface. Numbers above scale bars represent microns.

SmAP is widely distributed throughout adult male and female worms as determined by immunolocalization (Fig. 3A). Parasite muscle and parenchymal tissue stain clearly with anti-SmAP antibodies. Fig. 3B shows a higher magnification image of the periphery of an adult male section where the outer region of the tegument shows clear staining (arrow). Localization of SmAP by immunogold electron microscopy (Fig. 3C) confirms that the protein is distributed on the host-interactive tegumental membranes. Arrows in Fig. 3C point to some of the tegumental immunogold particles at the host parasite interface. Parasites treated with secondary antiserum alone demonstrate no tissue staining (data not shown).

### SmAP gene suppression

**Figure 4:**
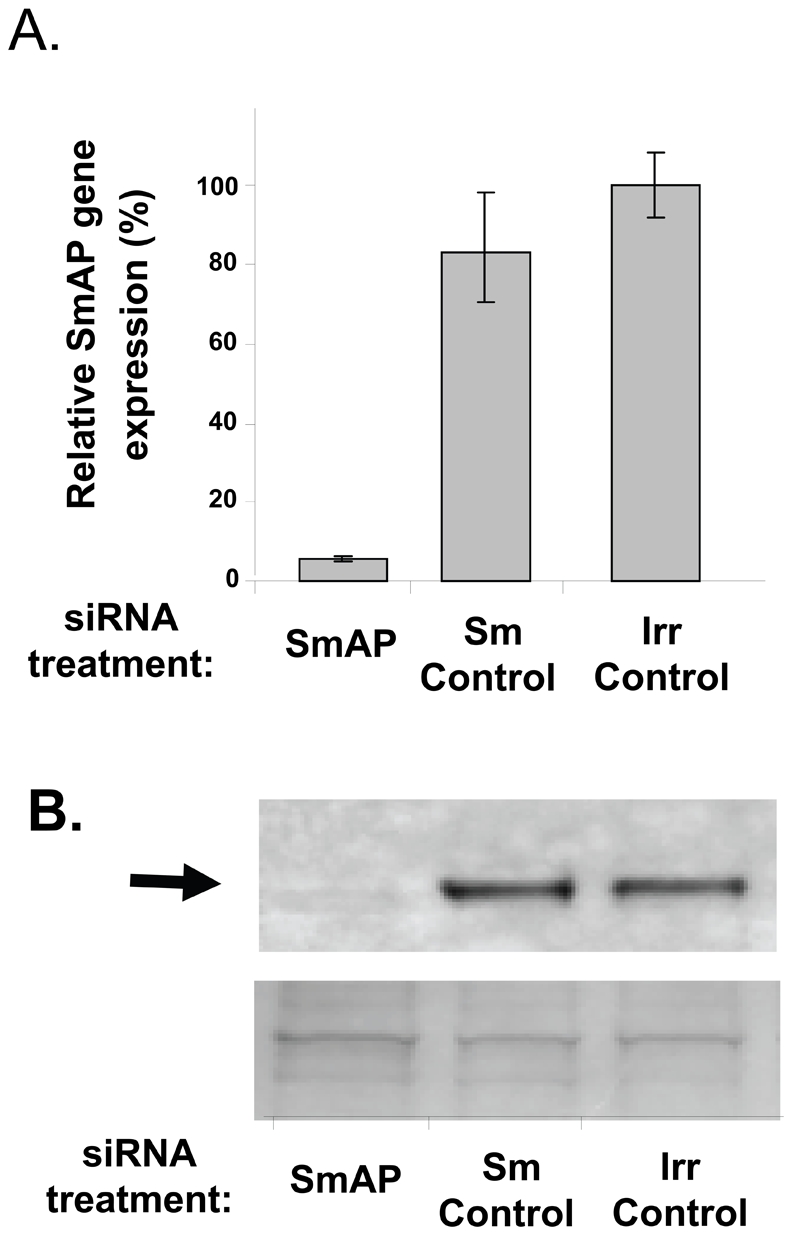
Suppression of SmAP gene expression by RNAi. A: Relative SmAP gene expression (mean±S.E.) in adult worms 10 days after treatment with SmAP siRNA or a control siRNA targeting another schistosome gene (Sm control) or an irrelevant control (irr control) siRNA. B: Detection by western blot of SmAP protein in adult parasite extracts 10 days after treatment with SmAP siRNA, a control siRNA targeting another schistosome gene (Sm control) or an irrelevant control (irr control) siRNA (top panel). The arrow indicates the greatly diminished level of SmAP protein seen in extracts of parasites targeted with SmAP siRNA (left lane). The bottom panel shows a strip of the gel stained with Coomassie blue to illustrate roughly equivalent protein loadings in each lane.

SmAP gene expression was suppressed in adult parasites *in vitro* by introducing a target specific siRNA using electroporation. Fig. 4A shows the specific and robust suppression of SmAP (∼95%), measured by qRT-PCR, 8 days after treatment. Western blot analysis demonstrates that this treatment also results in substantial diminution in SmAP protein production (arrow, Fig. 4B). The lower panel in Fig. 4B shows a fragment of a Coomassie Blue stained polyacrylamide gel, distant from the location of SmAP, to illustrate that all lanes contain roughly equivalent amounts of parasite protein.

**Figure 5:**
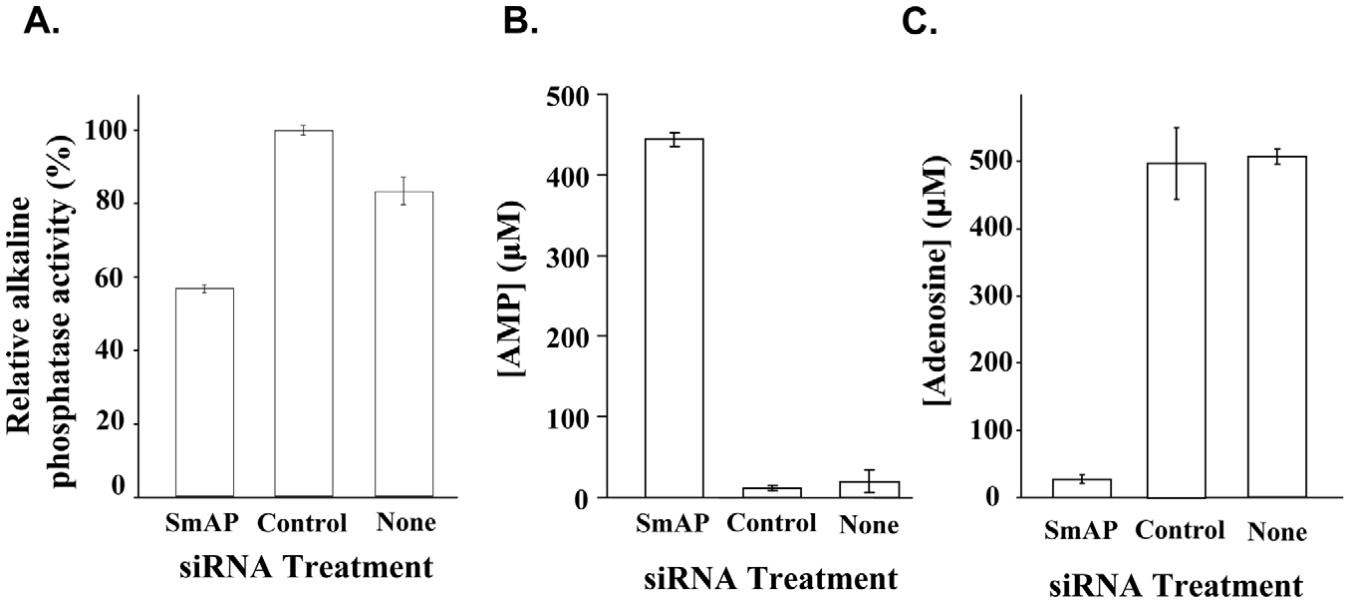
SmAP suppression phenotype. A. Relative alkaline phosphatase activity (mean±S.E.) displayed by live adult parasites 8 days after treatment with SmAP, control or no siRNA. B. AMP remaining (µM, mean±S.E.) in culture medium containing adult parasites 8 days after treated with SmAP, control or no siRNA, 40 hrs after the addition of 500 µM AMP. C. Adenosine generated (µM, mean±S.E.) in culture medium containing parasites treated with SmAP, control or no siRNA, 40 hrs after the addition of 500 µM AMP.

This robust suppression of SmAP does not result in any detectable change in adult parasite morphology or behavior. However, living adult parasites whose SmAP expression is suppressed by RNAi treatment, unlike controls, have a greatly diminished ability to cleave the chromogenic substrate, p-nitrophenyl phosphate (p<0.01, Fig. 5A). In addition, relative to controls, the SmAP-suppressed parasites are significantly impaired in their ability to cleave the nucleotide, AMP (p<0.01, Fig. 5B) and to generate the reaction product adenosine (p<0.01, Fig. 5C).

## Discussion

In this report we describe the cloning and characterization of a *Schistosoma mansoni* alkaline phosphatase (SmAP). Alkaline phosphatases (EC 3.1.3.1) are dimeric enzymes present in most, if not all, organisms. They catalyze the hydrolysis of phosphomonoesters with the release of inorganic phosphate. The SmAP cloned here is predicted to be a ∼60 kDa protein that possesses six potential N-linked glycosylation sites. This is in broad agreement with earlier work in which the enzyme was purified from Triton X-100 extracts of adult parasites following ConA agarose affinity chromatography and shown to be composed of ∼65 kDa glycosylated subunits [22]. Here we show that in adult schistosome extracts, anti-SmAP antibodies detect a protein of about this size by western blotting. Under non-reducing conditions an additional ∼120 kDa species is detected which likely represents the SmAP homodimer. Cysteine residues conserved in SmAP (boxed in Fig. 1) are probably important in protein dimerization. It appears that the 120 kDa molecule is functionally active since reaction product is detected at this site in gel slices incubated with alkaline phosphatase substrate. This is in agreement with the observation that enzyme active site residues and those coordinating metal ion binding are highly conserved in the predicted SmAP protein. No activity of the ∼60 kDa monomeric SmAP form is apparent. It has been reported in other systems that significant conformational changes occur during alkaline phosphatase dimerization that enhance thermal stability, metal binding and catalysis [23]. Thus alkaline phosphatases from several organisms, existing in a dimeric quaternary structure, are functional while their monomeric forms are inactive [23].

SmAP is highly expressed in the adult tegument as shown by immunofluorescence and immuno-electron microscopy and this concurs with previous work in which alkaline phosphatase activity was detected in worm sections on the external surface of the tegument [4], [24], [25]. Localization using immuno-EM shows that a majority of the immunogold particles appear to be distributed at the edge of surface pits which is consistent with a host interactive localization of this enzyme in the tegument. High alkaline phosphatase activity was previously reported in tegument enriched fractions of both male and female worms [26], [27], [28]. More recent tegumental proteomic analysis confirms that the protein is found in the schistosome surface membranes [29], [30] and is available for surface biotinylation [31]. Alkaline phosphatase activity was also previously detected in the material released from cultured schistosomula following phosphatidylinositol phospholipase C treatment, suggesting that some of the enzyme is phosphatidyl inositol anchored at the surface [32]. This is in keeping with SmAP sequence analysis which reveals the presence of a potential site for GPI-linked modification at residue S^492^. Immunological detection of SmAP reveals that the protein is also widely expressed in the internal tissues of the adult parasites and this corroborates earlier reports also detecting high levels of alkaline phosphatase enzyme activity internally [24], [33].

We have hypothesized that the function of schistosome tegumental SmAP is to bias host purinergic signaling pathways towards the generation of anti-inflammatory molecules such as adenosine [34]. In this work we show that living schistosomes can generate adenosine from exogenous AMP. This ability is effectively abolished when the expression of SmAP is suppressed following RNAi treatment. This result suggests that SmAP, a GPI-linked enzyme at the tegument surface, can access exogenous AMP and cleave it to generate adenosine. Extracellular adenosine is a potent immunosuppressant; it has been shown to be capable of dampening many facets of host immunity [35], [36], [37]. For instance, it can inhibit pro-inflammatory cytokine production and chemotactic responses of macrophages and monocytes as well as impairing macrophage proliferation, phagocytosis and lysosymal enzyme secretion [38], [39]. In addition, adenosine can inhibit reactive nitrogen species and reactive oxygen species production by monocytes/macrophages [40] and can impede lymphocyte adhesion and attenuate the proliferative and cytotoxic responses of activated T cells [41], [42]. Extracellular adenosine has been shown to inhibit adhesion of neutrophils to vascular endothelial cells and, at higher concentrations, to induce apoptosis of promyelocytes [43], [44], [45]. Adenosine can inhibit the generation of reactive oxygen species and the oxidative burst by immunostimulated neutrophils, as well as inhibit neutrophil degranulation and the generation of several inflammatory mediators [46]. Our data show that tegumental SmAP can generate adenosine and it seems likely that such an ability would benefit schistosomes by lessening the potential for inflammation and creating a less immunologically hostile, local environment for the parasites. Other pathogens, and some tumors, have also been shown to generate adenosine as a proposed immunosuppressant [36], [47]. Since SmAP can be efficiently suppressed without adverse impact on parasite viability in culture this suggests that the protein does not fulfill an essential function for parasites *in vitro* and is consistent with a role for this molecule *in vivo*.

In addition to SmAP, other phosphatase enzymes have been identified at the schistosome surface, including a phosphodiesterase and an ATPdiphosphohydrolase [48], [49]. Since SmAP suppression by itself is sufficient to effectively abolish the ability of schistosomes to cleave exogenous AMP, it is clear that these additional enzymes are not very important in the generation of adenosine through AMP cleavage. Since RNAi treatment results in SmAP gene knockdown but not knockout, it is likely that the minimal degradation of AMP seen in the SmAP suppressed group derives from residual SmAP protein and not from the action of other tegumental enzymes.

Since schistosomes cannot synthesize purines *de novo*, they must salvage these important biomolecules from the host [50]. It is possible that tegumental phosphatases, including SmAP, by dephosphorylating host molecules such as AMP to generate adenosine, play an important role in purine uptake in addition to their proposed role in immunomodulation highlighted here [51]. The higher relative expression of SmAP in adult female parasites lends weight to this notion.

Data reported here show that SmAP is expressed not only in the tegument but in internal tissues and in life stages outside the mammalian host, such as in sporocysts. Whether any adenosine generated in the intermediate snail host can act as an immunosuppressant is not clear since an effect of adenosine on invertebrate immune function has not been reported. In other systems alkaline phosphatases have been shown to play roles in a variety of metabolic processes including lipid absorption, connective tissue mineralization, and phosphate transport [52]. In addition to its proposed role in generating the immunosuppressant adenosine, it is possible that SmAP also fulfills some of these functions for schistosomes both in internal tissues and at the host-parasite interface.
